# Uncertainly Analysis of Two Types of Humidity Sensors by a Humidity Generator with a Divided-Flow System

**DOI:** 10.3390/s18020637

**Published:** 2018-02-21

**Authors:** Ling-Hsi Chen, Chiachung Chen

**Affiliations:** 1 Taichung District Agriculture Research and Extension Station, Changhua County 51541, Taiwan; chen52@tdais.gov.tw; 2 Department of Bio-industrial Mechatronics Engineering, National ChungHsing University, Taichung 40227, Taiwan

**Keywords:** uncertainty, humidity sensors, inverse calibration, Divided-flow system

## Abstract

Humidity measurement is an important technique for the agricultural, foods, pharmaceuticals, and chemical industries. For the sake of convenience, electrical relative humidity (RH) sensors have been widely used. These sensors need to be calibrated to ensure their accuracy and the uncertainty measurement of these sensors has become a major concern. In this study, a self-made divided-flow generator was established to calibrate two types of electrical humidity sensors. The standard reference humidity was calculated from dew-point temperature and air dry-bulb temperature measured by a chilled mirror monitor. This divided-flow generator could produce consistent result of RH measurement results. The uncertainty of the reference standard increased with the increase of RH values. The combined uncertainty with the adequate calibration equations were ranged from 0.82% to 1.45% RH for resistive humidity sensors and 0.63% to 1.4% for capacitive humidity sensors, respectively. This self-made, divided-flow generator, and calibration method are cheap, time-saving, and easy to be used. Thus, the proposed approach can easily be applied in research laboratories.

## 1. Introduction

Humidity measurement is an important technique for various industries. Humidity affects the air properties and materials that come into contact with air. The processes of manufacturing, storing, and testing are related to humidity [1]. Accurate humidity measurement is the key technology for preventing the condensation, corrosion and mold development, and thus, for enhancing the safety of the food, pharmaceutical, and chemical industries. Humidity measurement also contributes to how air-conditioners govern the thermal comfort of human beings [2]. Humidity levels affects the stomata openness of the leaves of plants, which then controls the functions of evaporation and photosynthesis [3].

Air humidity can be expressed as dew point temperature, absolute humidity and relative humidity. Relative humidity (RH) is the common term used to express the amount of the vapor contained in a moist air. Because they offer convenient and real-time measurement, electrical RH sensors have been widely used in various industries [4]. These sensors must be calibrated to ensure their accuracy. The uncertainty measurement of these sensors has become a major concern of users [5].

There are four types of humidity standard generator systems: fixed-point humidity systems, two-pressure humidity generators, two-temperature humidity generators, and two-flow humidity generators [4].

The fixed relative humidity point can easily be made with a saturated salt solution [6]. Several fixed RH points can be maintained with different saturated salt solutions. These then serve as the secondary standards to calibrate the humidity sensors. This method is easy to produce, convenient, and inexpensive, and can be used in research laboratories. However, the standard RH ranges are limited with these salt solutions. The equilibrate time required for the RH environment to reach the stable state is time-consuming. The uncertainty of humidity sensors calibrated by the saturated salt solutions have been evaluated in detail [7].

In the two-pressure method, an air stream at higher pressure than atmosphere air is saturated at a pre-set temperature and then be thermally expanded into a test chamber at that air pressure. The relative humidity is calculated by the pressure and temperature values of the test chamber. The uncertainty analysis of this kind of humidity generator has been introduced [8]. Heinonen [9] reported the measurement uncertainty in the temperature range from −40 °C to 77 °C, and the expanded uncertainty is ±0.08 °C for dew point temperature. Martins et al. [10] evaluated the uncertainty of the dew-point temperatures in this kind of two-pressure humidity generator using the GUM method and the Monte Carlo method and found that the measurement uncertainty ranged from ±0.1°C to 0.25 °C. The problems of testing and verification for the two-pressure system were discussed by Hardy [11].

The theory of the two-temperature method is to saturate the air with water vapor at a fixed temperature and then to increase the air temperature to a presetting value. Heinonen [12] discussed the development of a two-temperature humidity generator where the uncertainty of the dew point temperature was less than ±0.125 °C in the temperature range from −40 °C to 60 °C. Ahmed et al. [13] described the main parts of a two-temperature humidity generator to test the uncertainty of a chilled-mirror hygrometer and a thermos-hygrometer. In the RH range of 13–98%, the measurement uncertainty ranged from ±0.25% to 0.67% RH. El-Galid ad Ahmed [14] established two types of two-temperature humidity generators and found the measurement uncertainty of dew-point temperature in the range from ±0.09 °C to ±0.15 °C for the air temperature, ranging from −50 °C to 0 °C.

In the two-flow method, two streams are mixed in the test chamber to produce the different humidity levels. One stream is absolute dry gas and the other is saturated with water. The reference humidity in the test chamber is calculated from the flow rates of the two gas streams. The key of the technique is to control the ratio of the two flow rates. Takahashi and Kitano [15] proposed a new calibration of flow meters used in a divided flow humidity generator to enhance the mixing performance of this generator. Milosevic et al. [16] presented a method to establish a mixed-flow humidity generator. The uncertainty measurement of dew point temperature ranged from 0.2 °C to 0.5 °C. Dias [17] studied the uncertainty of the hygrometers in the lower humidity ranging from 2% to 30% at room temperature. The expanded uncertainties were in the range of 0.2% to 0.7%. In another report, a home-made divided-flow system was used to test the performance of humidity sensors [18]. The Hygropalm 2 hygrometer (Rotronic Inc., Bassersdorf, Switzerland) was served as standard humidity sensor. The standard uncertainty of this device was 0.3174% RH at the fixed RH value of 60.499% RH [18].

The accuracy of commercial electrical sensors is usually evaluated by the secondary standard humidity value. The dew-point temperature is the common standard. Recently, the measurement uncertainty of commercial electrical sensors has become a matter of concern [5]. The definition and evaluation method has been described in detail [19,20,21]. Huang [5] presented the uncertainty determination methods of the relative humidity, dew-point temperature, and mixing ratio in different humidity standard generators. Hardy [22] calculated the uncertainty of the relative humidity from dew-point temperature and air temperature, finding that as the air temperature ranged from −15 °C to −40 °C, and the accuracy of dew-point and air temperature was ±0.1 °C, and the total expanded uncertainty was in the range of 0.374% to 0.656%. Lin and Hubbard [23] reported the humidity uncertainties ranging from 0.6% to 6.6% when accuracies of dew-point and air temperature were ±0.3 °C and ±0.2 °C, respectively. The air temperature was in the range of −40 °C to 40 °C. Orlando et al. [24] calculated psychrometer properties of humidity measurements with different equations and software and found that the uncertainty of dew-point temperature measurement must be less than ±2 °C in order to ensure that the uncertainty of RH% is below ±1.0% RH. Lu and Chen [7] reported the measurement uncertainty of the relative humidity for two types of electrical humidity sensors calibrated by several saturated salt solutions. In the humidity range from 30% to 90% RH, the uncertainty ranged from 1.1843% to 1.1865%, and from 0.5710% to 0.5815% RH for resistive and capacitive humidity sensors, respectively. Mathioulakis et al. [25] calculated the relative standard uncertainties of the humidity ratio for different uncertainties of the air temperature, dew-point temperature, relative humidity, and pressures. The calculation of measurement uncertainty of relative humidity by the psychrometer method have also been presented [26]. Recently, novel devices of humidity sensors have been reported. Next generation capacitance sensing device using quartz crystals allows for humidity measurements in the uncertainty range below 0.2%. This high precision measurements using new switching sensing devices compensate temperature drift [27]. The NiO–SnO_2_ ceramic nanofibers have been fabricated and served as the humidity sensing element to observe the effect of relative humidity on electrical resistance of sensing element in the humidity ranged from 0 to 100% RH [28].

The two-flow humidity generator method is widely used in many testing and calibration laboratories. However, the cost is still expensive and this system is complicated, especially when it comes to precisely controlling the flow rates of dry and saturated stream. In this study, a self-made divided-flow humidity generator system was established to calibrate two types of electrical humidity sensors. A dew-point hygrometer was served as the reference standard. The measurement uncertainties of these humidity sensors were evaluated with the ISO GUM.

## 2. Sources of the Uncertainty of Humidity Sensors

### 2.1. Uncertainty of the Reference Standard

The RH values calculated from the dew point temperature, T_d_ and air temperature, T_a_ serve as the standard values to establish the calibration equations for resistive-type and capacitance-type sensors.

(1) $$RH=Pvs(T_{d})/Pvs(T_{a})$$

where Pvs(T_d_) is the saturated vapor pressure of T_d_ and Pvs(T_a_) is the air temperature T_a_.

(2) $$Pws=\exp[A_{1}^{}/T+A_{2}^{}+A_{3}^{}T+A_{4}^{}T_{}^{2}+A_{5}^{}T_{}^{3}+A_{6}^{}\mathrm{In}(T)]$$

The unit of T is Kelvin temperature

The uncertainty of the reference standard, u(RH_ref_) is calculated as follows:

(3) $$u_{ref}=u(RH_{ref})={\left(\frac{\partial RH_{ref}}{\partial T_{a}}\right)}^{2}u^{2}(T_{a})+{\left(\frac{\partial RH_{ref}}{\partial T_{d}}\right)}^{2}u^{2}(T_{d})$$

where u(T_a_) and u(T_d_) are the uncertainly of T_a_ and T_d_.

(4) $$\frac{\partial RH_{ref}}{\partial T_{a}}=-{\frac{Pvs(T_{d})}{Pvs\left(T_{a}\right)}}^{}*[-A_{1}^{}/T_{a}^{2}+A_{3}^{}+2A_{4}^{}T_{a}^{}+3A_{5}^{}T_{a}^{2}-A_{6}^{}/T_{a}^{}]$$

(5) $$\frac{\partial RH_{ref}}{\partial T_{d}}=\frac{Pvs(T_{d})}{Pvs\left(T_{a}\right)}*[-A_{1}^{}/T_{d}^{2}+A_{3}^{}+2A_{4}^{}T_{d}^{}+3A_{5}^{}T_{d}^{2}-A_{6}^{}/T_{d}^{}]$$

### 2.2. Uncertainty Due to Nonlinearity and Repeatability

The uncertainty U_non_ due to nonlinearity and repeatability is illustrated by manufacturers and is assumed to have a rectangular distribution. The numeric value for the capacitance-type sensor is a function of reading values of RH. The uncertainty u_non_ is calculated as follows:

(6) $$u_{non}=\pm U_{non}/2\sqrt{3}$$

### 2.3. Uncertainty Due to Resolution

For the uncertainty due to resolution of the sensor’s reading value, U_res_ is assumed to have a rectangular distribution. The uncertainty value due to resolution is estimated as follows:

(6) $$u_{res}=\pm U_{res}/2\sqrt{3}$$

### 2.4. Uncertainty Due to Temperature Variation and Hysteresis

The uncertainty measurement due to temperature variation and hysteresis is not mentioned by these sensors’ manufacturers. The effect of hysteresis for several humidity sensors was found to be insignificant [29]. The uncertainty measurement due to temperature variation and hysteresis was not considered in this study.

### 2.5. Uncertainty Due to Calibration Equation

The inverse method of calibration was used in this studied. The uncertainty of $RH_{cal}$ is calculated by the following Equation (7):

(7) $$u(RH_{cal})=s\sqrt{1+\frac{1}{n}+\frac{{\left(X_{i}-\bar{X}\right)}^{2}}{\sum_{}^{}(X^{2}{}_{i})-\sum_{}^{}{\left(X_{i}\right)}^{2}/n}}$$

where s is the stand deviation of the calibration equation, n is the number of measurements for the calibration equation, X_i_ is the RH values used to establish the calibration equation.

### 2.6. The Combined Standard Uncertainty

In this study, the uncertainty sources included reference standard, nonlinearity and repeatability, resolution and the calibration equation.

The combine standard uncertainty ($u_{c}$) can be estimated by the following:

(8) $$u_{c}{}^{2}=u^{2}{}_{ref}+u^{2}{}_{non}+u^{2}{}_{res}+u^{2}{}_{cal}$$

## 3. Materials and Method

### 3.1. The Divided-Flow System

**Figure 1:**
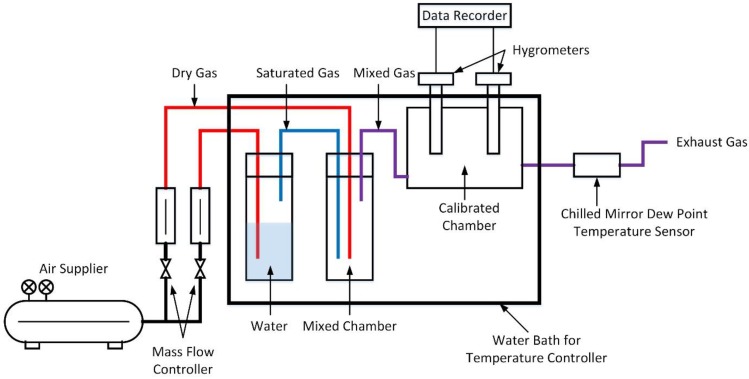
The schematic of a self-made divided-flow humidity generator.

The schematic of a self-made divided-flow humidity generator is shown in Figure 1. The entering dry gas was divided with mass flow controllers. The dry air and saturated air were mixed and then entered into a calibrated chamber. The total flow-rate was 3 L/min. Two types of electrical hygrometers were placed in the calibrated chamber for calibrating. The air flowed into a chill mirror dew temperature monitor. The relative humidity was calculated by the dew-point temperature and dry-bulb temperature measured by a chill mirror dew temperature monitor and served as the reference standard. The required humidity of mixed gas could be produced by adjusting the mass flow controller to change the ratios of dry and saturated air. A water bath was used as the temperature controller to ensure the isotherm state of testing.

Because the reference standard humidity values were measured by dew-point mirror monitor and were not computed by the flow-rates of saturated and dry air, no precise and expensive flow controllers were required. The cost of this system is acceptable for many industries and research institutes.

### 3.2. Sensors

The M4 chilled mirror monitor (General Eastern Instrument Inc., Wilmington, MA, USA) was used as the RH standard reference for calibration. The air temperature sensor used a platinum resistive thermometer. The accuracy of this thermometer was 0.1 °C after calibration. Two types of hygrometers, a THT-B11 resistive hygrometer (Shinyei Kaisha Co., Kobe, Japan) and a H51P-143A capacitive humidity transmitter (Vaisala Cor. Helsinki, Finland), were used to establish its calibration equations and calculate their measurement uncertainties. The specifications of these sensors are listed in Table 1.

### 3.3. Calibration to Standard Humidity System

The calibration of two types of electrical hygrometers at the temperatures ranging from 20 °C to 25 °C was carried out. The mass flow controllers were manually adjusted. As the humidity environment in the calibrated chamber was stable, the reading values of the resistive RH sensor, capacitive RH sensor, and dew-point temperature of a chilled mirror monitor were recorded. Then two mass flow controllers were adjusted to the next RH level. It took three to four hours to finish the whole calibration process from low to high RH level. Three repetitions were performed for the calibration.

### 3.4. Establishment of the Calibration Equation

The establishment of the calibration equation builds the relationship between the standard humidity values and the reading values of sensors. There are two statistical ways to establish the calibration equations: the classical method and inverse method. Because the predicted uncertainty of the inverse method is significantly lower than that of the classical method [7], the inverse method was used in this study.

(9) $$y_{i}=f(x_{i})$$

where y_i_ is the standard RH values and x_i_ is the reading values of sensors.

If y_i_ and x_i_ have a linear relationship, then

(10) $$y=b_{0}+b_{1}\times x$$

where b_0_ and b_1_ are constants.

If y_i_ and x_i_ have a polynomial relationship, then

(11) $$y=c_{0}+c_{1}\times x+c_{2}\times x^{2}+\ldots +c_{n}\times x^{n}$$

where c_0_, c_1_, …,c_n_ are constants.

The calibration equation was analyzed by using of SigmaPlot v12.2 (SPSS Inc., Chicago, IL, USA). The quantitative criteria to compare the fitting performance of calibration equations were the coefficient of determination *R*^2^ and standard of the estimated value *s*. The suitable order of the polynomial equation was tested by the *t*-value of the parameter values of each equation. The qualitative criteria are the residual plots. For a suitable equation, the data distribution of residual values should be a horizontal band centered on zero.

## 4. Results

### 4.1. Calibration Equations of the Resistive Humidity Sensor

**Figure 2:**
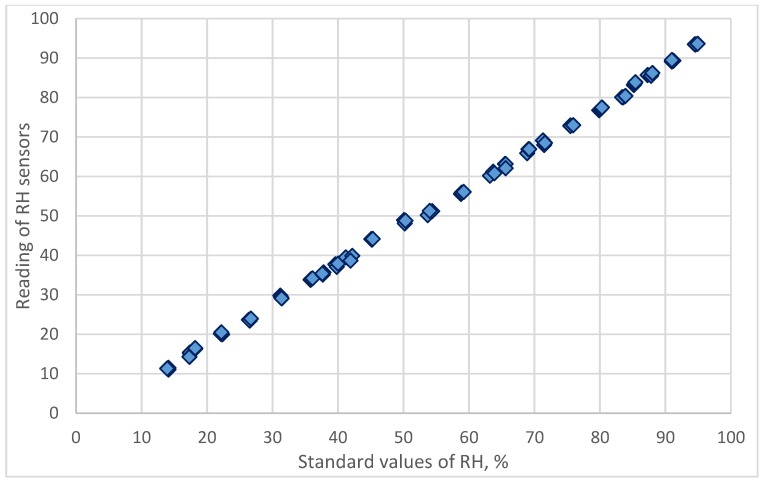
The relationship between reading values of resistive humidity sensor and the standard reference values calculated from the dew-point temperature and air temperature measured by a chilled mirror monitor.

The relationship between reading values of resistive humidity sensors and the standard reference values calculated from the dew-point temperature and air temperature measured by chilled mirror monitor are presented in Figure 2. The consistency of the data distribution of resistive humidity sensors showed the usefulness and reasonableness of this self-made divided-flow generator.

The calibration equation is established by regression analysis.

a. Linear equation
  (12) $$y=2.315065+1.00223\times x\;R^{2}=0.9992,s=0.8177$$

**Figure 3:**
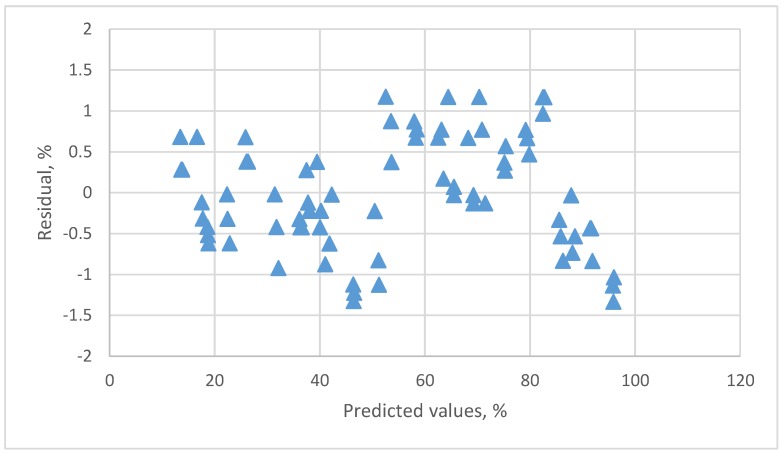
The residual plot of the linear calibration equation for resistive humidity sensor.

The residual plots of this linear equation are presented in Figure 3. A clear pattern is found. Although a high *R*^2^ was found, the residual plots indicated that the linear calibration equation was not a suitable equation.

b. 3rd order polynomial equation
  (13) $$y=4.276599+0.812626x+0.0045962x^{2}-0.000031528x^{3}\;R^{2}=0.9995,\;s=0.5565$$

**Figure 4:**
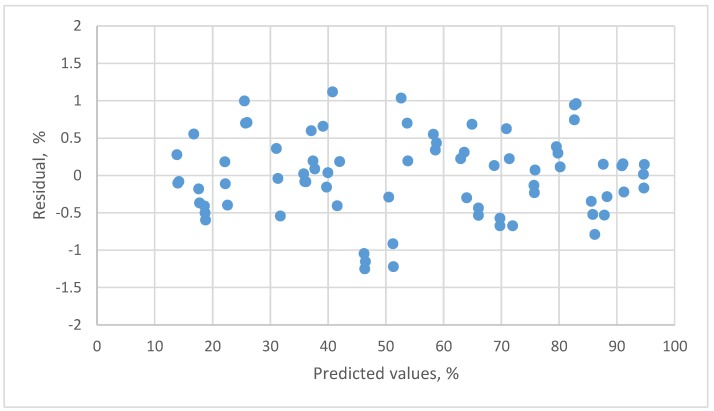
The residual plot of the 3rd order polynomial calibration equation for resistive humidity sensor.

The result for the second order polynomial equation presented a systematic pattern (not presented in the figure). The residual plot for the 3rd order polynomial equation is shown in Figure 4. By the *t*-tests of parameters and residual plots, this equation was adequate.

### 4.2. Calibration Equation of the Capacitive Humidity Sensor

**Figure 5:**
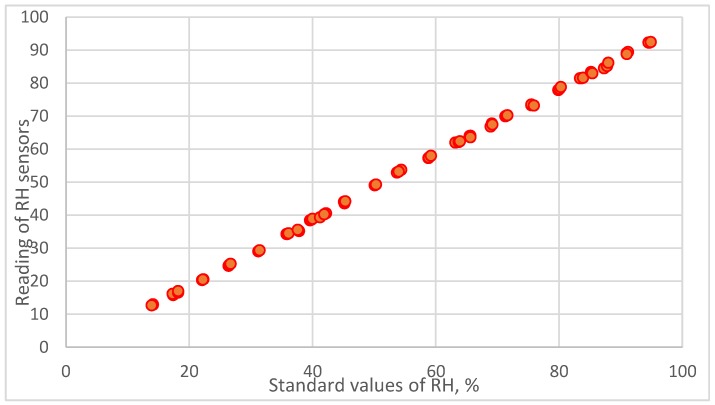
The relationship between reading values of capacitive humidity sensor and the standard reference values calculated from the dew-point temperature and the air, as temperature measured by a chilled mirror monitor.

The relationship between the reading values of capacitive humidity sensors and the standard reference values is shown in Figure 5. The consistency of the data distribution of this capacitive humidity sensor also revealed the usefulness of this self-made divided-flow generator.

a. Linear equation
  (14) $$y=1.19978+1.008537x\;R^{2}=0.9996,s=0.6320$$
b. 3rd order polynomial equation
  (15) $$y=0.991850+1.476546x-0.0012782x^{2}+0.0000102204x^{3}\;R^{2}=0.9998,s=0.3961$$

**Figure 6:**
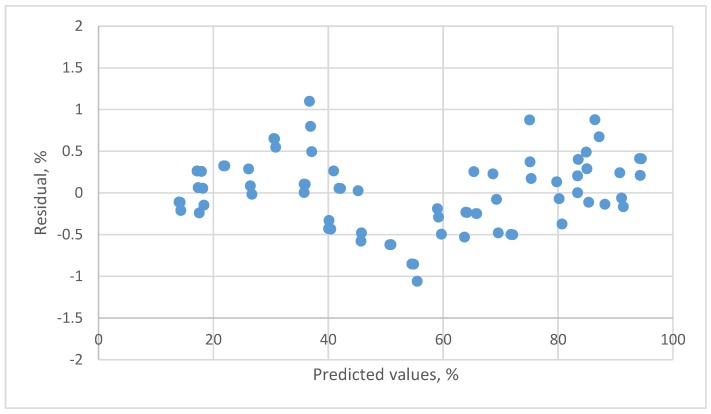
The residual plot of the linear calibration equation for capacitive humidity sensor.

The residual plots of the linear equation for the capacitive humidity sensor shown in Figure 6.

**Figure 7:**
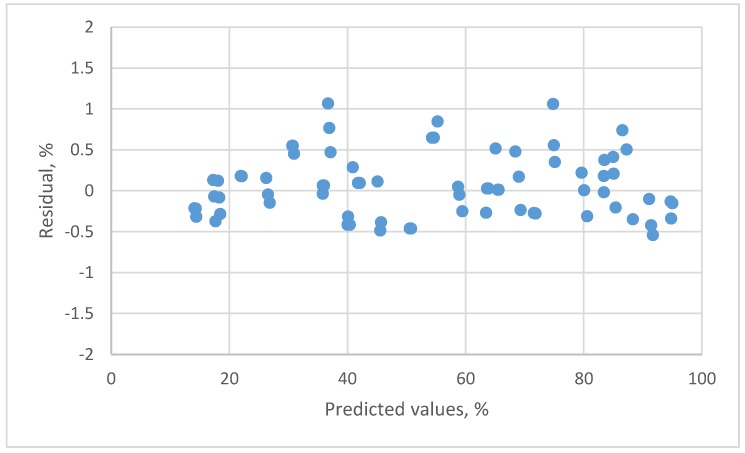
The residual plot of the 3rd order polynomial calibration equation for capacitive humidity sensor.

The clear pattern indicates that the linear equation was not a suitable equation. By the *t*-tests of parameters and residual plots (Figure 7), the 3rd order polynomial equation was shown to be an adequate calibration equation.

### 4.3. The Uncertainty of Reference Standard

**Figure 8:**
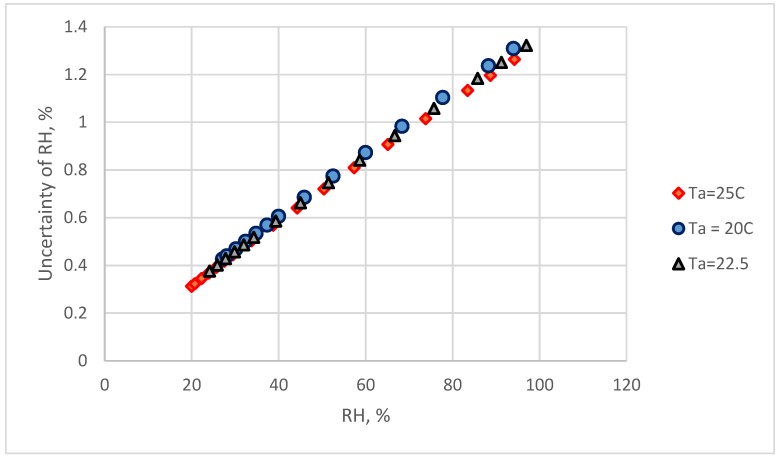
The relationship between relative humidity and reference humidity uncertainty values at different temperatures within ±0.2 °C accuracy of dew-point temperature.

The reference standard humidity is calculated by the dew-point temperature and air temperature. The uncertainty of reference standard humidity, u_ref_ is computed by Equations. (3) and (4). The u_ref_ values at different temperatures have the relationships with relative humidity (Figure 8). The u_ref_ is increased with the increase of RH values.

### 4.4. The Combine Standard Uncertainty of the Resistive Humidity Sensor

The uncertainty of the resistive humidity sensor for calibration equations, u_res_ was calculated by Equation (8):

(16) $$u_{res}\left(x\right)=s\sqrt{1.0133+{\left(x_{\mathrm{res}}-52.115\right)}^{2}/46403.4}$$

where s is the standard deviation of the calibration equation, and x_res_ is the reading RH values of the resistive sensor.

The type B uncertainty analysis for resistive humidity sensor is computed by Equations (6) and (7).

a. Uncertainty from nonlinearity and repeatability
  (17) $$u_{\mathrm{non}}=\pm U_{\mathrm{non}}/2\sqrt{3}=0.2886\%$$
b. Uncertainty from resolution
  (18) $$u_{\mathrm{res}}=\pm U_{\mathrm{res}}/2\sqrt{3}=0.02886\%$$

**Figure 9:**
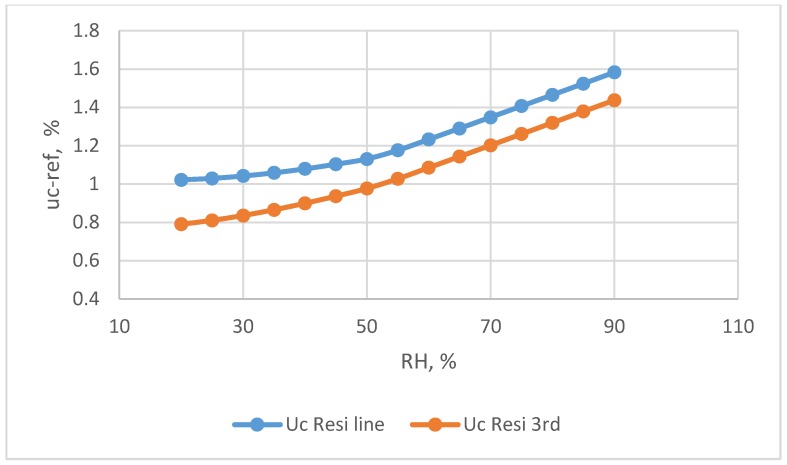
The combine uncertainty values for the resistive humidity sensors used two calibration equations.

The combined standard uncertainty of the resistive humidity sensor, u_c-res_, is calculated by Equation (9). The u_c-res_ value for the restive humidity sensors used two calibration equations are presented in Figure 9. At the 20%, 55%, and 90% levels of the observed humidity, the uncertainty values were 1.022%, 1.176%, and 1.583%, respectively, when using the linear calibration equation and 0.79%, 1.027%, and 1.437% when using the 3rd order polynomial calibration equation, respectively. The results indicated that a suitable calibration equation can decrease the uncertainty significantly. Because the reference standard uncertainty, u_ref_, reaches higher values in high RH ranges (Figure 8), the u_c-res_ was > 1.2% when the RH > 70%.

### 4.5. The Combined Standard Uncertainty of the Capacitive Humidity Sensor

The uncertainty of the capacitive humidity sensor, u_cap_ for calibration equation was calculated by Equation (8):

(19) $$u_{c}{}_{ap}\left(x\right)=s\sqrt{1.0133+{\left(x_{\mathrm{cap}}-52.79067\right)}^{2}/45661.5}$$

where s is the standard deration of the calibration equation, and x_cap_ is the reading RH values of the capacitive sensor.

The type B uncertainty analysis for the capacitive humidity sensor is computed as follows:

- Uncertainty from nonlinearity and repeatability
  (20) $$u_{\mathrm{non}}=\pm U_{\mathrm{non}}/2\sqrt{3}=1/3.4641\left(1.0+0.008RH\right)$$
- Uncertainty from resolution
  (21) $$u_{\mathrm{non}}=\pm U_{\mathrm{non}}/2\sqrt{3}=0.02886\%.$$

**Figure 10:**
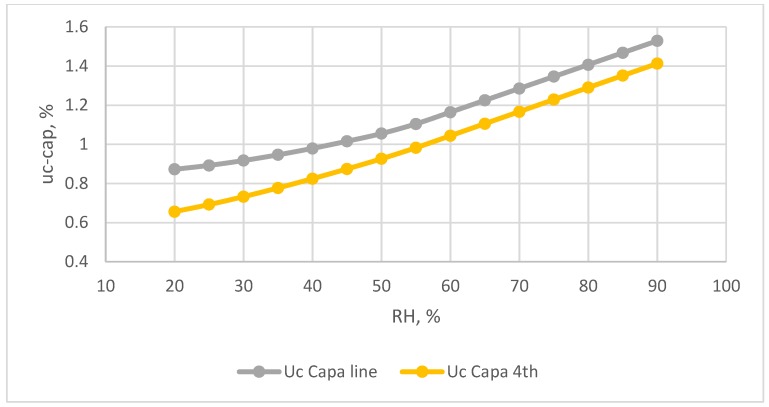
The combine uncertainty values for the capacitive humidity sensors used two calibration equations.

The combined standard uncertainty of the capacitive humidity sensor, u_c-cap_, as calculated by Equation (9), is shown in Figure 10. At the relative humidity values of 20%, 55%, and 90% levels of the observed humidity, the combined standard uncertainty values are 0.873%, 1.105%, and 1.530% using the linear calibration equation and 0.656%, 0.983%, and 1.413% for the 3rd order polynomial equation, respectively. The combined uncertainty obtained from the 3rd order polynomial equation is significantly lower than that of the linear equation.

## 5. Discussion

**Figure 11:**
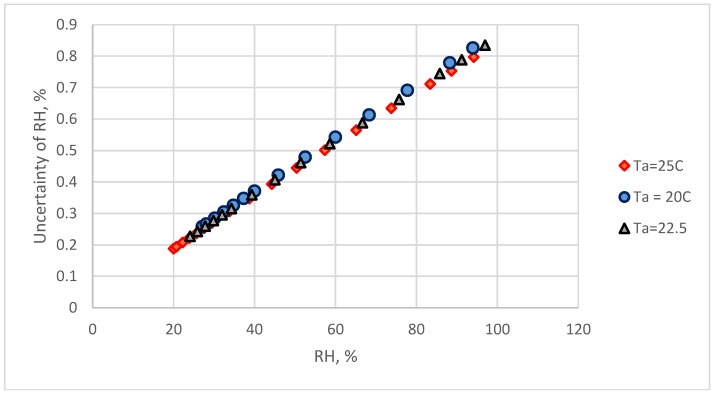
The relationship between relative humidity and reference humidity uncertainty at different temperatures within ±0.1 °C accuracy of dew-point temperature.

**Figure 12:**
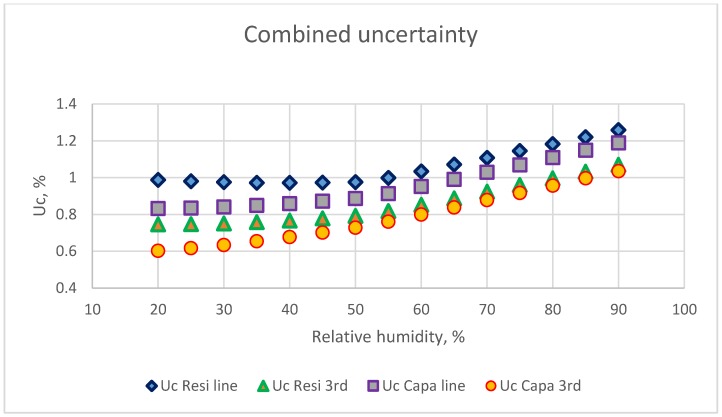
The combine uncertainty value for two types of humidity sensors used two calibration equations. The accuracy of dew-point temperature was within ±0.1 °C.

The main sources of the combined uncertainty were the standard reference and calibration equations. In this study, the reading values of a commercial chilled-mirror monitor were used as the standard reference. The accuracy of this dew-point temperature was ±0.2 °C, according to the specifications of the manufacture. For a dew-point temperature monitor with ±0.1 °C accuracy, the u_ref_ calculated by Equations (3)–(5) are shown in Figure 11. The u_ref_ value was < 0.7% when the RH value < 80% and less than 1.0% in the RH range higher than 80%. In this condition, the combined standard uncertainty for the two calibration equations and for two types of electrical humidity sensors are presented in Figure 12. The u_c-ref_ could be < 1.2% and u_c-cap_ < 1.05% at 90% RH. At 70% RH, u_c-ref_ could be < 1.0% and u_c-cap_ < 0.8%.

A previous study reported the uncertainty of humidity sensors at 60% RH, as evaluated by a home-made divided-flow generator [18]. The estimated uncertainty was 0.3174% RH. The standard uncertainty of the standard hygrometer was 0.22%, based on the reports of manufacturers. However, the combined uncertainty of their standard hygrometer that calibrated by the humidity standard generator systems was not reported.

In another works, two types of electrical humidity sensors were calibrated by several saturated salt solutions [7]. The combined uncertainty with adequate calibration equations at observed humidity levels of 30%, 60%, and 90% were 1.200, 1.208, and 1.202%, with a resistive sensor and 0.603, 0.605, and 0.613% with a capacitive sensors, respectively. The resistive electrical sensors results of this study were better than those of a previous study [7]. However, the combined uncertainty of the capacitive humidity sensors in this study were higher than those of the previous study [7]. The results could be explained by the different manufacturers and reference standard values.

Orlando et al. [24] mentioned that if the uncertainty of the dew-point temperature was less than ±2 ℃, then the RH uncertainty was below ±1.0%. This was confirmed by the results of this study. The measurement uncertainty of two types of electrical hygrometers was < 1.5% RH. When the cover factor equaled to 2, the expanded measurement uncertainty was < 3% RH. Thus, the proposed approach could meet the performance requirements for application in most of the industries [1].

In this study, the uncertainty of the reference standard increased with the increase of RH value. Therefore, the combined measurement uncertainty of the two types of electrical hygrometers was > 1.0% RH when the RH reading values > 70%. The uncertainty of the humidity environment produced by saturated salt solutions were < 0.55% [30]. However, the equilibrate times for the saturated salt solutions are in the range of 8 h to 12 h [6]. It takes several days to finish the several points’ calibration by a fixed-point humidity system. In this study, this calibration works was finish within several hours. It is a time-save work. The self-made calibration system in this study can be easily established in most of laboratories. The cost of this system is inexpensive. The key technique decreasing the measurement uncertainty is to use a more accurate dew-point temperature monitor. The calibration system and the establishment of the calibration equation developed in this study could be easily applied in industry laboratories.

## 6. Conclusions

In this study, a self-made divided-flow generator was established to produce a humidity environment for the calibration of two types of electrical humidity sensors. The dew-point temperature and air dry-bulb temperature of a chilled mirror monitor was measured to calculate the relative humidity value. This value then served as the standard reference humidity for calibration. The uncertainties of two types of electrical humidity sensors were evaluated and compared.

The consistency of the data distribution between the reading values of resistive and capacitive humidity sensors and standard reference values showed the usefulness of this divided-flow generator. The uncertainty of the reference standard increased with the increase of RH values. The combined uncertainty of the adequate calibration equations were ranged from 0.82% to 1.45 % RH for the resistive humidity sensor and 0.63% to 1.4% for the capacitive humidity sensor, respectively. When we used a more accurate dew-point temperature monitor, the combined uncertainty of the two types of electrical humidity sensors could be reduced to 1.0% RH. Because the self-made divided-flow generator and calibration method are cheap, time-saving, and easy to be used, therefore, the proposed approach can easily be established and applied in a research laboratories.

## Figures and Tables

**Table 1:** Specification of the humidity sensors.

	Dew Point Temperature	Resistive Humidity Sensor	Capacitive Humidity Sensor
**Sensing element**	Chilled mirror sensors	Macro-molecular Resistive element	Hamicap Capacitive-type
**Measuring range**	−80~85 °C 0.003~100% RH (Relative humidity)	0~99% RH	Thin-film polymer element 0~100% RH
**Nonlinearity and repeatability**	±0.05 °C	1.0% RH	(1.0 + 0.008* Reading value)% RH
**Resolution**	0.1 °C	0.1% RH	0.1% RH
**Accuracy**	Dew point temperature ±0.2 °C Air temperature ±0.1 °C (After calibration)	±2.0%, 20~90% RH	±1% RH, 0~90% RH
